# Mechanical ventilation for amyotrophic lateral sclerosis/motor neuron disease

**DOI:** 10.1590/S1516-31802010000200015

**Published:** 2010-03-04

## Abstract

**BACKGROUND:**

Amyotrophic lateral sclerosis, also known as motor neuron disease, is a fatal neurodegenerative disease. Without mechanical ventilation, death from respiratory failure usually follows within two to five years of the onset of symptoms.

**OBJECTIVES:**

To examine the efficacy of mechanical ventilation (tracheostomy and non-invasive ventilation) in improving survival, on disease progression and quality of life in amyotrophic lateral sclerosis.

**SEARCH STRATEGY:**

We searched The Cochrane Neuromuscular Disease Group Trials Specialized Register (December 8 2008), The Cochrane Central Register of Controlled Trials (The Cochrane Library Issue 4, 2008), MEDLINE (January 1966 to December 2008), EMBASE (January 1947 to December 2008), CINAHL Plus (January 1937 to December 2008), and AMED (January 1985 to December 2008). We also searched for ongoing studies on clinicaltrials.gov.

**SELECTION CRITERIA:**

Randomised and quasi-randomised controlled trials involving non-invasive or tracheostomy assisted ventilation in participants with a clinical diagnosis of amyotrophic lateral sclerosis.

**DATA COLLECTION AND ANALYSIS:**

Four authors independently selected studies for assessment. All authors extracted data independently from the full text of selected studies and assessed the risk of bias in studies that met the inclusion criteria. We attempted to obtain missing data where possible.

**MAIN RESULTS:**

Two randomised controlled trials involving 54 participants receiving non-invasive ventilation were identified and included. Incomplete data were published for one study and we contacted the trial authors who were not able to provide the missing data. Therefore the results of the review were based on a single study of 41 participants. The study showed that the overall median survival in the whole cohort after initiation of assisted ventilation was significantly different between the non-invasive ventilation and standard care groups (P = 0.0062) with a median survival for the non-invasive ventilation group patients of 48 days longer than the standard care group participants. Non-invasive ventilation significantly improved survival and quality of life in the subgroup with normal to moderately impaired bulbar function. Non-invasive ventilation did not prolong survival in patients with poor bulbar function although it showed significant improvement in the mean symptoms domain of the sleep apnoea quality-of-life index but not in the Short Form-36 quality of life mental component summary score.

**AUTHORS’ CONCLUSIONS:**

Evidence from a single randomised trial of non-invasive ventilation in 41 participants suggests that it significantly prolongs survival and improves or maintains quality of life in people with ALS. Survival and some measures of quality of life were significantly improved in the subgroup of people with better bulbar function, but not in those with severe bulbar impairment.

**FURTHER INFORMATION:** Centro Cochrane do Brasil Rua Pedro de Toledo, 598 Vila Clementino — São Paulo (SP) — Brasil CEP 04039-001 Tel. (+55 11) 5579-0469/5575-2970 http://ww.centrocohranedobrasil.org.br/

**This section was edited under the responsibility of the Brazilian Cochrane Center**

**The full review is available (free access) from:** http://cochrane.bvsalud.org/portal/php/index.php?lang=pt

## COMMENTS

Amyotrophic lateral sclerosis is a serious disease for which no specific treatment is currently available. Respiratory insufficiency is the leading cause of death and therefore any measures that address this problem should be greeted enthusiastically. The present review analyzed the advantages of mechanically-assisted ventilation and concluded that noninvasive methods of ventilation can benefit patients by increasing survival time and improving quality of life. The outcomes were better for patients with moderate bulbar impairment than for individuals with large lesions at this site.

Although the present study involved a relatively small patient sample, it is valuable given the rareness of the disease and the fact that the investigation included well-devised and complete studies. The study findings contribute towards medical practice by highlighting a serious problem and the benefits of proper management, thus indicating the need for further clinical studies in this largely unexplored field.

